# Exploring dendritic cell subtypes in cancer immunotherapy: unraveling the role of mature regulatory dendritic cells

**DOI:** 10.48101/ujms.v129.10627

**Published:** 2024-04-12

**Authors:** Oscar Badillo, Liam Helfridsson, Jenni Niemi, Mats Hellström

**Affiliations:** Department of Immunology, Genetics and Pathology, The Rudbeck Laboratory, Uppsala University, Uppsala, Sweden

**Keywords:** Dendritic Cell, Immunotherapy, mregDC

## Abstract

Dendritic cells (DCs) possess a specialized function in presenting antigens and play pivotal roles in both innate and adaptive immune responses. Their ability to cross-present antigens from tumor cells to naïve T cells is instrumental in generating specific T-cell-mediated antitumor responses, crucial for controlling tumor growth and preventing tumor cell dissemination. However, within a tumor immune microenvironment (TIME), the functions of DCs can be significantly compromised. This review focuses on the profile, function, and activation of DCs, leveraging recent studies that reveal insights into their phenotype acquisition, transcriptional state, and functional programs through single-cell RNA sequence (scRNA-seq) analysis. Additionally, the therapeutic potential of DC-mediated tumor antigen sensing in priming antitumor immunity is discussed.

## Introduction

The dendritic cells (DCs) are professional antigen-presenting cells located throughout the body and function as sentinels for danger. DCs play a crucial role in maintaining a delicate equilibrium between immunity and tolerance (1), a duality that can pose a double-edged sword in the context of cancer. Irrespective of their subset, DCs require an activating signal to trigger a terminal differentiation process called ‘maturation’, shifting DCs from an antigen accumulation mode to an antigen presentation mode (2). Upon encountering a proinflammatory stimulus, mature DCs actively promote immunity, aligning their response with the precise nature of the stimulus. Conversely, in the absence of such stimulation, DCs tend to foster tolerance. To evoke an effective anticancer response, antigen-accumulating DCs necessitate an appropriate activating signal (3, 4).

DCs play a key role in the immunoregulation against tumors and have the potential to revolutionize the field of immunotherapy. The latest years have armed us with expanding knowledge about DC subtypes, activation, function, and potential in antitumor treatment (5, 6). Therefore, this review aims to provide an overview of DC phenotype acquisition, transcriptional state, and functional programs in steady state and cancer, with a special focus on the central nervous system (CNS) and mature regulatory DCs (mregDCs). Furthermore, the current knowledge is related to the role of DCs in Tertiary Lymphoid Structures (TLS), and immunotherapy.

### Dendritic cell subtypes

In humans, DCs are commonly categorized into four subgroups: conventional dendritic cells 1 and 2 (cDC1 and cDC2), monocyte-derived dendritic cells (moDCs), and plasmacytoid dendritic cells (pDCs) (identified by CD123+ cells). More recently, a subset of DC has been discovered, identifying a population of cells that exhibit morphological similarities under exposure to various stimuli. These cells also express several key cytokines and transcription factors responsible for controlling DC development and diversification. Confirmation of the cDC subsets and further subclassification has been achieved using single-cell RNA sequencing (scRNA-seq). CD141/BDCA-3+ cDC1 and CD1C/BDCA-1+ cDC2 have been subdivided into DC2 and DC3, while DC4 represents CD1C−CD141−CD11C+ DCs resembling monocytes (7–9). Two transcriptional clusters of cDC2s in human blood that differently express B and T lymphocyte attenuator (BTLA) receptor can be best distinguished phenotypically as CD5+CD163–CD14– cDC2s and CD5–CD163+CD14+ cDC2s. DC3s in the blood, on the other hand, were confirmed to be the immediate precursors of tissue inflammatory DCs that develop via a specific pathway activated by granulocyte-macrophage colony-stimulating factor (GM-CSF), independently of common DC precursors (CDPs), and give rise to DCs with a unique ability to prime naive CD8+ T cells into tissue-homing CD103+ T cells (10). Human genetic studies further showed that cDC1s and cDC2s differentiate along a common trajectory distinct from DC3s (11). Additionally, DC6 comprises interferon-producing pDCs (7).

In mice, two major subsets of DCs known as conventional cDC1 and cDC2 can be distinguished by cell surface markers: cDC1 is characterized by MHC-II+, CD11c+, CD8+, CD103+, and CD11b-, while cDC2 expresses MHC-II+, CD11c+, CD8-, and CD11b+ (12). These subsets rely on distinct transcription factors for their development. While both cDC1 and cDC2 depend on GM-CSF for differentiation, cDC1 relies on IFN regulatory factor (IRF) 8 and Basic Leucine Zipper ATF-Like Transcription Factor 3 (BATF3). In contrast, cDC2 additionally relies on IRF4 and Zinc finger E box binding homeobox 2 (ZEB2) (13) Table 1.

**Table 1:** Markers and genes profile of peripheral DC subpopulations in the steady state humans, and mice.

Subtype	Marker	References	Species
cDC1	XCR1, CADM1, CLEC9A, CD26, CD11c, CD64, MHCII/HLA-DR, IRF8, CD8α, CD11, CD26, CD70, CD80, CD86, CD103, CD141, CLEC9A	(20, 21)	Human
	XCR1, CADM1, CD24, CD26, CD11c, MHCII, IRF8, Clec9a, Cadm	(20, 22)	Mouse
cDC2	CD1c CD11, CD40, CD123, CD172a, CD303, ZEB2, FLT3 Irf4	(20, 21, 23)	Human
	CD172a, CD11b, CD26, MHCII/HLA-DR, CD24, CD206, CD135, PDL1, Irf2, Irf4	(20, 23, 24)	Mouse
pDC	CD11c, CD8a, PDCA-1, Bst2, Ly6d, CD303, CD68, CD317, CD304, CD123, BDCA-4, TLR7, CLECSF1, CD69, CLEC13A	(20, 21)	Human
moDC	Siglech, Bst2, Ly6c1, Cd4, Ccr9, If17rb, Notch1, Epha2, Grm8, Kira17, Zfp521, Ets1, Kik1, Bink, Dch1, Sh3bgr CD209, CD206, CD303, CD207, Zbtb46, CD317	(20)	Mouse
mregDC/DC3	CD16/32, CD172a CCR7, CD74, ID2, GPR183, CCL22, IL4l1, CD40, TXN, IL1B, IL-15, IL-23A, IDO1, LAMP3, CCR7	(25, 26)	Human
	Ccr7, Fscn1, Il4i1, Socs2, Relb, Cd40, Cd80, Reib, Cd83, Cd274, Pdcd1/g2, Cd200, Fas, Aldh1a2, Socs1, Socs2, Ccr7, Myo1g, Cxcl16, Icam1, Fscn1, Marcks, Marcksl1	(22)	Mouse

Only major DC subpopulations, cDCs, pDCs, moDCs, and mregDCs, are presented in the table. All the pathologies are excluded, and only studies showing the DC markers in steady state are included.

The cDC1 population remains a pivotal contributor to the initiation of CD8+ T cell-mediated tumor immunity. This ability is attributed, at least in part, to their capacity for processing and cross-presenting antigens on MHC-I molecules. Upon antigen presentation, the activation of DC1 is triggered, resulting in the production of interleukin (IL)-12 and their capability to traffic from the tumor bed to draining lymph nodes (dLNs). Additionally, it is plausible that these migrating cells may transfer tumor antigens to dLN-resident DCs, offering another pathway for antigen cross-presentation to T cells via both MHC class I and class II molecules (14, 15). On the other hand, cDC2s are commonly associated with antigen presentation on MHC class II molecules and the subsequent stimulation of CD4+ T cell responses.

During migration, DCs undergo a phenotypic transformation, maturing with increased expression of MHC-II and costimulatory markers such as CD80/86, CD40, and OX40L (16, 17). This maturation process enhances the cytokine production of DCs, including IL-12, IL-4, and TGF-β, which, in turn, modulates the activation and differentiation of T helper 1 (Th1), Th2, or regulatory T cells, respectively (18, 19). Additionally, DCs express regulatory markers such as CD200 and PD-L1/2, which inhibit maturation and the proinflammatory response, effectively suppressing and regulating the immune response (16, 17).

### The DC3 and mregDC states

Recent studies have highlighted the distinctive developmental, transcriptomic, phenotypical, and functional features of DC3s, delineating them as a unique subset of cells (9, 10). However, there remains extensive work to precisely define the discrete differentiation pathways and molecular states emerging under diverse inflammatory conditions. It is plausible that different cues and various maturation or activation states may give rise to distinct DC3 developmental profiles, expanding the spectrum of DC states within the DC3 subset, especially in inflamed settings.

The term ‘DC3’ is increasingly employed in various studies to denote inflammatory DCs, identifying a cellular cluster expressing a gene program linked to immune cell activation, observed in both murine and human tissues. Additionally, DC3s are found in both intratumorally and in dLNs, marked by the expression of CCR7 and LAMP-3 proteins (33), and reduced conventional cDC transcripts such as CD1c, FCER1A, CLEC10A, CLEC9A, and CADM1 (34).

**Figure 1:**
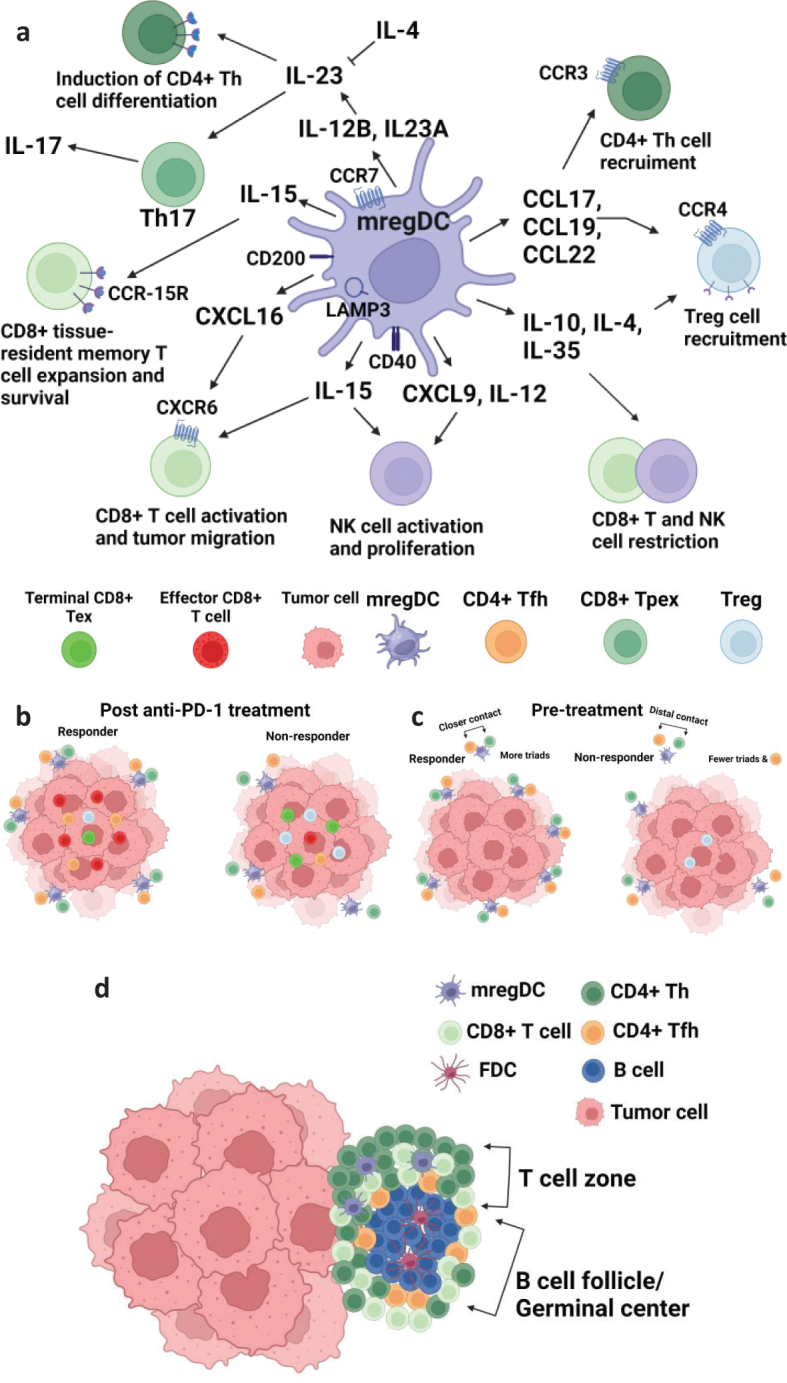
The cross-talk mregDCs in the TIME. The mregDCs are present in various tumors and tissues, sparking diverse research into their characteristics and shedding light on their role in tumor immunity. The function of mregDCs is intricately linked to the TIME, where cytokines and chemokines play a dual role, either activating or inhibiting their antitumor immunity (a). Through interaction with CD4+ T cells, mregDCs can enhance antitumor capacity, stimulate B cells, and express costimulatory molecules, thereby facilitating the recruitment and differentiation of CD4+, CD8+ T cells, and NK cells. Conversely, mregDCs can impede antitumor immunity by aiding in Treg recruitment while limiting responses from CD8+ T and NK cells. The mregDCs engage in cross-talk with CD4+ Th cells through stimulation of the TCR-MHC and the CD28-CD80/CD86 complexes. Increased expression of CD40, CD83, CD86, PD-L1, and CCR7 enhances their capacity to prime CTLs (b). Stimulation of mregDCs with minP1 and anti-PD-1 enhances their numbers and antigen presentation capabilities, leading to increased triads comprising CD4+ CXCL13+ Th cells and closer contact with mregDCs. This results in a superior immune response, including enhanced differentiation of progenitor exhausted (Tpex) into effector CD8+ T cells and heightened their infiltration, resulting in tumor regression, thereby improving overall survival and clinical relevance (c). Undergo a dynamic process, mregDC can migrate to the tumor site from peripheral lymph nodes or TLSs. TLSs encompass a B cell follicle/germinal center housing follicular DCs and CD4+ CXCL13+ Tfh cells. In the T cell zone, mregDCs coexist alongside CD4+ Th and CD8+ T cells (d). Created with BioRender.

This ‘DC3’ cluster shares several transcripts with ‘mature’ DCs, enriched in immunoregulatory molecules (mregDCs) recently described (22). The mregDC cluster characterizes a molecular state acquired by both cDC1s and cDC2s upon encountering or internalizing cell-associated antigens, exhibiting specialized antigen presentation programs and associated with regulatory, immunogenic, and migratory gene programs (Figure 1a). Previously, this specific molecular state was identified through bulk transcriptomic analysis of cDC1s and cDC2s migrating to the dLNs, characterized as migratory cDCs (35, 36). Intriguingly, scRNA-seq demonstrated the induction of both the mregDC and the ‘migratory’ DC state in genetically modified DCs capable of migration to the dLNs (22). The down regulation of canonical cDC1 and cDC2 gene transcripts correlates with the downregulation of these genes during DC maturation, as previously reported (35, 36). Notably, despite the reduced mRNA expression of canonical cDC1 and cDC2 markers upon antigen uptake and Toll-like receptor activation, corresponding surface proteins such as XCR1 or CD172a remain detectable on mregDCs even after their migration to the dLNs, underscoring the importance of using CITE-seq, or similar techniques, to distinguish DC subsets based on surface protein expression.

Taken together, these findings suggest that mregDCs, also known as CCR7+LAMP3+ DCs, represent a distinct molecular state induced in cDC1s, cDC2s, and potentially inflammatory DC3s, exhibiting unique differentiation pathways upon exposure to stimuli and demonstrating a unique capacity. However, the distinction between mregDCs and DC3s in T cell activation remains unclear, and the specific details are not yet understood, highlighting the need for further research to gain a better understanding of the molecular system that dynamically regulates their development and maintenance. In this review, we refer to migratory DCs or CCR7+ LAMP3+ cells as the mregDC population.

### Dendritic cells in cancer

Several research groups have systematically mapped tumor-infiltrating DCs without relying on predefined protein markers. These studies have successfully characterized the profiles of DCs infiltrating various tumor types, including melanoma (37), hepatocellular carcinoma (33), head and neck squamous cell carcinoma (38), non-small cell lung cancer (NSCLC) (22), cutaneous squamous cell carcinoma (39), ovarian cancer, breast cancer, and colorectal cancer (34, 40). Despite employing distinct tissue dissociation protocols, scRNA-seq technologies, and bioinformatics methodologies across these studies, the results consistently revealed a distinct set of profiles among tumor-infiltrating DCs (41, 42). Furthermore, DC populations in human and mouse share a gene and protein expression patterns (22, 34, 43) underscoring conserved traits across species despite tumor heterogeneity.

The presence of DCs in tumors is pivotal for anticancer immune responses. Tumors described as ‘immune deserts’ show almost no response to immunotherapy and lack T cell infiltrates, indicating the absence of an ongoing immune response. These tumors often lack DCs, possibly contributing significantly to the unresponsiveness (44). In scenarios where the tumor immune microenvironment (TIME) lacks sufficient inflammation, there is also a reduced likelihood of DCs undergoing maturation or generating antitumor T cells.

Studies in human cancer have demonstrated the formation of clusters or ‘triads’ comprising T cells (CD4+ and CD8+) alongside DCs within the tumor (45–48) and found both in dLN and the TIME (Figure 1b, c). These cells exhibit immune costimulatory and regulatory properties, recognized as crucial elements in regulating the immune response (22, 49). Intratumorally, DCs direct T cells toward differentiation into effector, memory, or exhaustion pathways, and recent evidence suggests that migratory CCR7+ DC related with DC1 and DC2 subpopulation might play separate roles in the development and exhaustion of T cells (50).

### Dendritic cells in the central nervous system

The DCs are not typically found in normal brain parenchyma but are instead primarily present in vascular-rich compartments such as the choroid plexus and meninges (51, 52). In nonlymphoid tissues like the brain, DCs display distinct characteristics compared to those in other tissues Table 2. DCs do not cross the intact glia limitans. They either remain for a short period, ultimately dying or leaving the CNS. However, they are crucial for inducing local activation of T cells in the perivascular, postcapillary venules, or the leptomeningeal space of the CNS (53). In-depth analysis utilizing mass cytometry, t-SNE mapping, and FlowSOM-guided clustering in whole brains from healthy 8-week-old mice has revealed the presence of cDC1, cDC2, and pDC subsets in the CNS (24). Notably, cDC2s were relatively more abundant than cDC1s in brain tissue. However, during pathological conditions such as chronic inflammatory diseases, acute infections, neurodegeneration, and cancer, DCs have been observed to migrate to the brain and spinal cord via afferent lymphatics or high endothelial venules (54). For instance, following a stroke, a significant increase in DCs, particularly cDC1 cells, is noted in the CNS (55).

**Table 2:** Markers and genes profile of DC subpopulations in CNS steady state and tumors.

Subtype	Marker	References	Description
cDC1	CD11, CD26, CD70, CD80, CD86, CD103, CD141, CLEC9A, CD24, CD135, CD117	(21, 24)	Steady state CNS
	XCR1, CLEC9A, CADM1, CD26, CD141, CD13, CD226, CD72, CD56, CD103, CD272, CD218a, CD205, CD24, XCR1, CD103, CD371	(27–29)	Melanoma brain and leptomeningeal metastases, glioblastoma
cDC2	CD1c, CD11, CD40, CD123, CD172a, CD303, ZEB2, FLT3, CD24, CD206, CD135	(21, 24)	Steady state CNS
	HLA-DPB1, HLA-DQB1, CD1E, CD1C, CLEC10A, HLA-DQA2, CD1A, CD207, CEACAM3, CD196, CD267, CD117, IL1B, ZEB2, CD14, CD9, CD64, CD354, CD41, CD209a, CD11b, CD172a, CD301a, CD326, CD301b, CD117, CD300LG, CD43, CD62L, Ly6C	(27–29)	Melanoma brain and leptomeningeal metastases, Glioblastoma
	CCL3L1, AF1, FCER1A, SGK1, MAFB, LST1, YWHAH, S100A4, CCL2, MS4ASA	(30)	Breast cancer brain metastases
Monocyte DC	LYZ, HLA-DRB1, TIMP1, S100A11, CXCL8, IL1B, PTGS2, S100A9, S100A8, MMP19	(29)	Melanoma brain and leptomeningeal metastases
	CD14, CD1c, C1QA, CD141, IL1B, CLEC10A CD34, CD303, CD304, CCR7	(21, 28)	Glioblastoma
	CST3, HLA-DPA1, HLA-DQB1	(31)	Primary central nervous system lymphoma
Plasmacytoid DC	CD1c, CD123, CD135, CD303, CD304, CD172	(21, 24)	Steady state CNS
	CLEC4C, IRF7, TCF4, GZMB	(29)	Melanoma brain and leptomeningeal metastases
	IL3RA, LILRA4, CD123, CD45RA, CD304, CD36, SiglecH, CD45R-B220, CD8b, CD4, CD317, CD31, CD38	(27)	Glioblastoma
	PTGDS, GZMB, CLIC3, TPM2, JCHAIN, ITM2C, IRF7, LILRA4, CCL4, TCL1A	(30)	Breast cancer brain metastases
MregDC/DC3	LAMP3, TXN, R8CN1, BIRC8, CDM3, MARCK8L1, R831, CCR7, CS17, MU81, BAMSN1, IL4I1, DU8P5, IL82, IDO1, TMEM176A, ILTR, BCL241	(32)	Head and neck squamous cell carcinoma
Migratory DC	IA-IE, CD274, CD80, CD86, CD200, CD1d, CD357, CD25, CD150, CD95, CD63, CCR7, LAMP3, SAMSN1, CD273, CD86, CD71, CD127, FSCN1, PDL1/2 ICAM1, CCR7, IL4R, IDO1/EBi3, TIM3, IL32, IL15, IL12, CXCL16, CCL19, CCL17, CCL22, CD273, Socs2	(27–29)	Glioblastoma, melanoma brain, and leptomeningeal metastases

The specific role of DCs in the context of CNS malignancies is still under investigation. Current studies indicate a complex interplay between DCs, microglia, macrophages, T cells, and tumor cells within the TIME. One proposed role for DCs in this setting involves the recognition and presentation of tumor antigens either within the brain or in the tumor-draining deep cervical lymph nodes to elicit coordinated T cell-mediated responses (54). However, glioma stem cells have been observed to differentiate DCs into tolerogenic phenotypes with low expression of costimulatory molecules. In cell culture assays, these DCs exhibited high proliferative, invasive, and migratory abilities, potentially impacting tolerogenic T cell responses (56). Droplet-based scRNA-seq of brain and leptomeningeal metastases of melanoma revealed the presence of pDC, cDC1, cDC2, DC3, macrophages, moDC, and myeloid-derived suppressor (MDSC)-like cells. A higher proportion of pDCs, cDC2s, DC3s, and moDCs was associated with a positive therapy response, while fewer pDCs and more cDC2s were linked to a poor response (29). In both human and murine gliomas, upregulated pDCs with high expression of MHC-II were correlated with lower survival rates (57). In addition, scRNA-seq of brain metastases from breast cancer illustrated the presence of tolerogenic DCs that are derived from cDC1s and cDC2s in the TIME. Notably, these tolerogenic DCs expressed high levels of negative immune regulators such as IDO1, LGALS3, LGALS9, and NECTIN2 and possessed the ability to inhibit the activation of CD8+ T cells by recruiting Treg cells (30). These reports suggest that DCs with a tolerogenic profiles have been associated with primary and metastatic brain tumors, but certain patterns of subpopulations of DCs have been associated with better prognosis. Further studies of the DC populations are needed to refine the prognostic and therapeutic relevance.

### Dendritic cells and tertiary lymphoid structures

The characterization of TLSs has significantly enhanced our understanding of the TIME, highlighting its potential to foster an immunostimulatory milieu alongside its well-documented immunosuppressive aspects. TLSs, resembling ectopic lymphoid aggregates similar to secondary lymphoid organs, consist of a B cell follicle encircled by follicular-like dendritic cells (antigen-presenting cells of mesenchymal origin), within a T cell zone housing CD4+ follicular Th cells, CD8+ T cells, DCs, and high endothelial venules (58). These structures mimic germinal centers, a long-recognized feature in tumors, in addition to the presence of less organized lymphoid aggregates (44). TLSs likely serve as organized LN-like structures facilitating T cell activation and expansion via tumor-associated DCs, indicating that the activation of T cells by DCs is not confined solely to secondary lymphoid organs like the dLN but is also a critical component within the TIME itself. This phenomenon likely extends beyond the TLS and involves antigen presentation by DCs dispersed throughout the TIME and intratumorally (Figure 1d). Essentially, they contribute to the constitution of a TLS-like structure adjacent to the tumor, effectively resembling a lymph node in close proximity, thereby potentially enhancing a robust immune response. This might explain why subpopulations of DCs are frequently observed in peritumoral regions, interacting with other infiltrating immune cells such as CD4+ and CD8+ T cells (46, 58–60).

Clinical studies consistently link positive responses to checkpoint therapies with the presence of TLSs in the TIME (61–64). Notably, in a mouse model, TLS formation was observed proximal to the meninges rather than within the tumor mass, showing their existence in Glioblastoma Multiform (GBM) patients. Tumor biopsies and surgical resection of gliomas often lack meningeal tissue, contributing to the oversight of TLSs in this cancer type. However, a limited number of cases exhibited intratumoral or peritumoral TLSs (65). Intriguingly, immature TLS-like lymphoid aggregates were only identified in 8% of evaluated GBM cases (66).

An intriguing aspect involves mregDCs and their role in the antitumor response within TLSs. Studies have indicated the prevalence of mregDCs in peritumoral regions surrounding the tumor and their close proximity to T cells (59, 60), relating the potential dominance of mregDCs within TLSs.

In the case of Head and Neck Squamous Cell (HNSCC) tumors, the presence of TLSs was confirmed, with DCs located in the T cell zone in direct contact with T cells, while exhibiting LAMP-3 expression related with mregDC phenotype. Patients displaying immune profiles of TLSs with LAMP-3+ DCs exhibited a better prognosis for survival and demonstrated a more favorable response to anti-PD-1 immunotherapy, showing a higher degree of complete response (58). Notably, mregDCs are predominantly found in the peritumoral area, aggregating around various immune cells, particularly CD4+ and CD8+ T cells. Immunohistochemistry (IHC) analysis in esophageal squamous cell carcinoma (ESCC) and primary cutaneous melanoma (PCM) revealed LAMP-3+ DCs infiltrating the peritumoral area and aggregating around CD8+ and CD25+, and OX40+ T cells, respectively (67, 68). These peritumoral mregDCs showed a positive correlation with CD8+ T cell infiltration, which was associated with a better prognosis for survival (59, 60).

### Prognostic relevance of mregDCs for immunotherapy

The role of mregDCs in the TIME underscores their significance in regulating immune responses Table 3. Notably, mregDCs maintain close contact with Tregs, expressing markers like CCR7 and CD127, which uphold a mature phenotype and foster Treg proliferation (32). Furthermore, mregDCs significantly enhance Treg infiltration into tumors by being among the highest producers of chemokines like CCL17 and CCL22 (67).

**Table 3:** Prognostic value of mregDCs in cancer.

Cancer	Method	Samples	Species	Markers	Findings	Name	Ref.
ESCC	IHC	80 surgical treated without pre-opt care	Human	LAMP-3	Infiltrating LAMP-3+ DCs correlated with intratumoral CD8+ T cells, which correlated with a favorable prognosis	LAMP-3+ DCs	(60)
PCM	IHC	82 primary tumor samples	Human	LAMP-3	LAMP-3+ DCs aggregated around activated T cells & together provided a strong prognosis for survival	LAMP-3+ DCs	(59)
HNSCC	RNA seq & cohorts with multivariate analysis	TCGA-HNSCC database & human tumors	Human	LAMP-3	LAMP-3+ DCs are found within TLSs, and these are correlated with better prognosis	LAMP-3+ DCs	(58)
	Mass cytometry & bulk RNA seq	Primary & metastatic tumors	Human	*Cd274, Pdcd1lg2, Cd40, Cd80, Cd86, Cd200, Il4i1, Il4r, Il12b, Ccr7*	mregDCs enrich Tregs by expressing CCR7 & CD127. They also produce IL-23, which increases IL-17 production by Th17 & exhausted CD8+ T cells, which has a favorable prognosis	mregDCs	(32)
LLC & Colon26	Flow cytometry	Tumor samples from minP1, anti-PD-L1, minP1+anti-PD-L1 treated & untreated	Mice	CCR7+, CD11b+, CD11c+, CD14-, Ly6c-, MHC-II+, from lineage-negative cell population: CD3-, CD19-, Ly6G	All treatments increased the number of intratumoral mregDCs, while being correlated with an expansion of intratumoral progenitor CD8+ T cells while decreasing terminally exhausted CD8+ T cells. Enhancing the prognosis & favoring survival	mregDCs	(69)
	scRNA seq	Tumor samples from minP1, anti-PD-L1, minP1+anti-PD-L1 treated & untreated	Mice	*Fscn1, IL12b, Ccl22*	minP1 upregulated the presentation of MHC-II antigens on mregDCs. The treatments exerted better prognosis & survival	mregDCs	(69)
TNBC	scRNA seq	Tumors from retrospective clinical trials	Human	*Ccl19, Ccr7, Lamp3, Fscn1*	CCL19+ DCs indicated better response to anti-PD-1 and were also expanded in responsive tumors. Experienced CD8+ T cells were predictive for response to anti-PD-1, and these cells were increased in CCL19+ DC-enriched tumors	CCL19+ DCs	(70)
LLC1	scRNA seq	Tumors from two clinical trials	Human	*Ccr7, Il12b, Ccl22*	PGE antagonism decreased tumor growth, by reducing the production of CCL17 & CCL22 by mregDCs, which lowers the tumor-infiltrating Tregs, which benefits inflammation	mregDCs	(67)
HCC	scRNA seq & staining	Tumor lesions from responders & non-responders of anti-PD-1	Human	*Cd274, Ccr7, Ccl22, Birc3, Ido1, Il4i1, Lamp3*	Cellular triads consisting of mregDCs, CXCL13+, CD4+ Th & progenitor CD8+ T cells were found in responding tumors, and deemed critical for the differentiation of progenitor CD8+ T cells into effective antitumor CD8+ T cells in response to PD-1 blockade	mregDCs	(46)
NSCLC	scRNA seq & CITE-seq	Naïve & tumor-bearing lungs	Human & mice	*Cd274, Pdcd1lg2, Cd200, Cd40, Ccr7, Il12b, Cd80, Cd86, Cd83, Relb, Fas, Socs1, Socs2, Aldh1a2*	IL-4 blocking resulted in increased production of IL-12 by mregDCs, which enhanced the T cell activation, and expansion of IFNγ+, CD8+ effector T cells resulting in reduced tumor growth	mregDCs	(22)

IHC: immunohistochemistry; ESCC: esophageal squamous cell carcinoma; PCM: primary cutaneous melanoma; HNSCC: head and neck squamous cell carcinoma; LLC: Lewis lung carcinoma; HCC: Hepatocellular carcinoma; NSLC: Non-small cell lung cancer; TCGA: The Cancer Genome Atlas; TNBC: triple-negative breast cancer.

However, the impact of the mregDC-Treg axis on the TIME during immunotherapy varies across cancer types. For example, in HNSCC, mregDCs express high levels of the IL23A and IL12B genes, resulting in the production of IL-23 protein, which leads to the generation of IL-17 by Th17 cells and exhausted CD8+ T cells. This phenomenon correlates with a more favorable prognosis (32). Conversely, increased expression of genes encoding CCL17 and CCL22 has been associated with poorer prognoses in various human cancers, suggesting the negative impact of the mregDC-Treg axis. This effect is regulated by the PGE2-EP2/EP4 pathway, and its inhibition reduces Treg tumor infiltration, potentially indicating a positive prognosis (67).

Studies have linked the presence of mregDCs with favorable prognoses for survival and lower metastatic frequencies (59, 60, 68). For instance, in ESCC and PCM, tumors without metastases exhibited higher frequencies of LAMP-3+ DCs compared to metastatic tumors, indicating a correlation between mregDCs and nonmetastatic tumors. Similarly, increased abundance of LAMP-3+ DCs was associated with decreased metastasis beyond the initial dLNs in melanoma tumor samples (68).

Moreover, in patients with GBM, neoadjuvant anti-PD-1 therapy upregulated the genes related with mregDC profile; however, the increase of activated markers CD40 and CD86 remained quite small, leading to suboptimal antigen presentation and an insufficient antitumor response (47). In mice, studies of colon and Lewis Lung Carcinoma (LLC) models demonstrated that a treatment combining anti-PD-L1 and a stimulatory high-mobility group nucleosome binding domain 1 (HMGN1) peptide called minP1 led to tumor regression, immunological memory, and increased survival rates by stimulating mregDCs (69).

Further investigations targeting mregDCs’ secretion of IL-12 have shown promise in improving T cell stimulation. Blocking IL-4, which suppresses IL-12 production, in NSCLC mouse models augmented cytokine production by CD4+ T cells activated by mregDCs, resulting in reduced tumor growth and an increased expansion of IFNγ+TNF+CD8+ T cells (22).

### Dendritic cell vaccination

The growing understanding of the immune system in genetics and functionality in both the CNS and the TIME has led to a notable increase in clinical trials exploring immunotherapy for primary brain tumors. Various immunotherapeutic strategies, including ICIs like PD-1/PD-L1 or CTLA-4, oncolytic viruses, cancer vaccines, cytokines, and adoptive cell therapies, have been extensively studied and documented in extracranial malignancies (71). However, the emerging challenge is to comprehend the CNS toxicities associated with immunotherapy among patients diagnosed with primary brain tumors, considering their unique physiology and associated challenges. The insufficient responses observed with current immunotherapies have prompted the focus on identifying potential targets that significantly impact the course of the antitumor response.

Due to their vital role as sentinel cells capable of recognizing various TAAs, dendritic cell vaccination (DCV) has emerged as an intriguing approach to elicit a robust immune response against cancer (17). DC-based immunotherapy has shown promise as a therapeutic approach in various systemic cancers, as indicated by several studies (72, 73). For instance, targeting DCs via an agonistic antibody targeting CD40 has demonstrated efficacy in inducing DC activation in cancers (34, 74). Conversely, the use of ICI like anti-PD-1/PD-L1 has displayed a positive impact on DCs, fostering an antitumor response (75).

The success of DC therapies in treating other cancers has sparked growing interest in using DCVs against CNS malignancy, prompting numerous preclinical studies to evaluate their efficacy and feasibility. For instance, Siesjö et al. demonstrated that pre-immunization with mutagen-treated rat glioma N32 cells resulted in the rejection of subsequent subcutaneous injections (76). A similar experimental model highlighted the effectiveness of DCVs in triggering cytotoxic CD8+ T cell-mediated antitumor immunity, evidenced by increased infiltration of CD8+ T cells in the TIME (77). Furthermore, data from a phase III clinical trial with DC autologous tumor lysate vaccine suggest prolonged overall survival (78). However, an external-matched control that was used as a data from a randomized trial is warranted. Subsequent studies have presented variations in methodologies, including alternative selections for the pulsed antigen, diverse routes of vaccine administration, and incubation methodologies, each with varying effectiveness in eliciting an antitumor response (79–82). Despite methodological differences, these studies collectively demonstrated the potential of DCVs to provoke an antitumor response. Over time, various research groups have endeavored to identify the optimal methodology and adjuvant therapies that could enhance the efficacy of DCVs in combatting GBM in preclinical models.

## Concluding remarks

Recent advancements reveal that DCs function not only in LNs but also within the TIME. The widespread adoption of single-cell transcriptomic technologies in the past few years has yielded an explosion of information of the molecular states of all cells within solid tumors, including dendritic cells. Immunotherapy, beyond PD1/PD-L1 and CTLA4 blockade, is emerging as a promising treatment for various tumors. Most investigations are focused on exploiting T-cell functions directly, but interventions targeting other cell types, particularly targeting DCs, have great potential. DC-based therapies confer a distinct advantage, especially in low-immunogenic tumors, by bolstering antitumor T cell responses. These studies, both in cancer models and patients, consistently rediscover DCs and T cells for their contributions to predictive signatures for successful immunotherapy and favorable disease outcomes.

Moreover, the modulation of immunoregulatory mediators expressed by DCs fine-tunes T-cell activation thresholds and regulates immune responses. However, despite achieving successful DC migration and eliciting tumor-specific responses, their effectiveness might be compromised due to their significant heterogeneity, plasticity, and modulation influenced by the TIME, particularly under the impact of immunotherapies.

In summary, DCs play a pivotal role in presenting tumor antigens and initiating adaptive immune responses. The evolving understanding and precise characterization of tumor DC states are revealing molecular targets to exploit DC biology for therapeutic purposes. A comprehensive grasp of DC plasticity concerning signals from the TIME promises fresh perspectives for antitumor therapy, refining current treatments and devising innovative targeted strategies.
